# Stakeholders' views and opinions on existing guidelines on “How to Choose Mental Health Apps”

**DOI:** 10.3389/fpubh.2023.1251050

**Published:** 2023-11-22

**Authors:** Wishah Khan, Bertina Jebanesan, Sarah Ahmed, Chris Trimmer, Branka Agic, Farhana Safa, Aamna Ashraf, Andrew Tuck, Kelsey Kavic, Sapna Wadhawan, Maureen Abbott, Omair Husain, Ishrat Husain, Muhammad Akhter Hamid, Kwame McKenzie, Yuri Quintana, Farooq Naeem

**Affiliations:** ^1^Centre for Addiction and Mental Health (CAMH), Toronto, ON, Canada; ^2^Department of Psychiatry, University of Toronto, Toronto, ON, Canada; ^3^Mental Health Commission of Canada, Ottawa, ON, Canada; ^4^Department of Paediatrics, University of Toronto, Toronto, ON, Canada; ^5^Scarborough Health Network, Scarborough, ON, Canada; ^6^Department of Medicine, Harvard University, Boston, MA, United States; ^7^Beth Israel Deaconess Medical Center, Boston, MA, United States

**Keywords:** mobile Apps, mental health, digital health, guidelines, evaluation

## Abstract

**Background:**

Mental health Applications (Mhealth Apps) can change how healthcare is delivered. However, very little is known about the efficacy of Mhealth Apps. Currently, only minimum guidance is available in Assessment and Evaluation Tools (AETs). Therefore, this project aims to understand AET developers' perspectives and end users' experiences and opinions on “how to choose a Mhealth App”.

**Objective:**

The primary objectives were: (1) obtaining stakeholder's opinions and experiences of development and use of AETs for Mhealth Apps, their weaknesses and strengths, and barriers in their implementation of Mhealth Apps; (2) the experiences of App users, their analyzation and, obstacles in the use of apps; and (3) to quantify themes related to choosing a Mhealth App.

**Methods:**

This qualitative study, used a sampling method to recruit six stakeholders (one App developer, two AET developers, an individual with lived experience of mental health illness, and two physicians) who were interviewed using a topic guide. These were examined by researchers (CT, WK, & FN) using thematic content analysis. Additionally, an anonymous online survey of 107 individuals was conducted.

**Findings:**

Our analyses revealed six main themes: (a) needs and opportunities; (b) views on Mhealth apps; (c) views & opinions on AETs; (d) implementation barriers; (e) system of evaluation and; (f) future directions. The first key concept was, all stakeholders agreed that Apps could significantly impact mental health and that end-users were unaware of mental health AETs and Apps. Secondly, due to commercial interests, end-users reliability of App evaluations requires clear conflict-free guidelines. Thirdly, AETs should be evaluated and developed through a rigorous methodology. Finally, stakeholders shared insights into future developments for AETs and Mhealth Apps. Additionally, online survey respondents chose a “health professional” as their preferred source of guidance in selecting a Mhealth app (84%) and best suited to develop guidelines (70%).

**Conclusion:**

The interviews and survey highlight the need for Mhealth Apps to be regulated and the importance of health professionals' engagement in the implementation process. Similarly, without well-defined roles for App evaluations within the health care system, it is unlikely that AETs will have wider spread use and impact without risk.

## Introduction

Social distancing and changes in practice around COVID-19 have forced health providers worldwide to provide services through online platforms, thus acting as a catalyst to raise awareness, interest, and uptake of mobile health applications (mHealth Apps) (1). In addition, some countries have reported changes in legislation and policy to promote telemedicine (2, 3). As a result, the demand for mHealth Apps is strong. A recent public survey found that 76% of 525 respondents would be interested in using their mobile phones for self-management and self-monitoring of mental health if the service was free (4). In a similar survey of physicians' attitudes toward mobile health (MHealth), most expressed hope that technology could be very effective in their clinical practice (5).

There are currently more than 10,000 Apps created explicitly for mental or behavioral health (6) out of 318,000 health Apps (7). The primary function of these Apps is to target the medical disease or disorder in terms of prevention, management, and treatment of the health issues. However, as the numbers of the mHealth Apps increase, so do the apprehensions surrounding the safety and effectiveness of these Apps (8). Considering medical devices and pharmaceuticals undergo a thorough assessment to be licensed, the equivalent evaluation is beginning to be expected of mHealth Apps. In healthcare, this is necessary to guarantee any reputable technology's effective and safe operation (7).

Mental health applications (Mhealth Apps) may play an essential part in the future of mental health care (4) by making mental health support more accessible (9). However, there is insufficient evidence for the effectiveness of these Apps. A recent publication reported that only 3.4% of Mhealth Apps were included in research studies to justify their claims of effectiveness, with most of that research undertaken by those involved in developing the App (10). It has been observed that a clinically relevant App for people living with depression becomes unavailable and deleted from App stores every 2.9 days (11). Similarly, App stores require regular updates, making it challenging to keep track of a quickly evolving field (12). Furthermore, people generally stop using a Mhealth App if they are not equipped with any guidance from a clinician (13). Along with a study reporting that within 7 days of downloading an App, over 56% of users uninstall them (14). This mix of potential and problems means there must be clear guidelines on “How to choose a Mhealth App”.

Mental health interventions and Mhealth Apps broadly vary significantly in their use. Therefore, evidence-based guidelines that have been established for mental health interventions do not apply to Apps. Presently, there is very limited regulation on the growth and reporting of Mhealth Apps, from their effectiveness, side effects, privacy and security, reporting, and scientific examination (15). However, due to these factors, the need to regulate Mhealth Apps increases (16). So far, only the FDA has approached a form of regulation with regulatory guidelines [the Digital Health Software Precertification (Pre-Cert) Program]. This program recognizes the unique and rapidly changing aspects of mHealth Apps and aims to streamline the regulatory oversight of software-based medical devices (17).

Several Assessment and Evaluation Tools (AETs) (e.g., frameworks, guidelines, rating systems, or App libraries) have been developed internationally (18–22). However, these initiatives are not without issues. For example, the NHS Apps Library, which assessed Apps against a defined set of criteria, was released but quickly rolled back due to public outcry following news that highlighted privacy and security gaps in many of the Apps (23). In addition, many AETs rely on expert consensus, which can be opaque and difficult to understand for both users and clinicians (24). There are also significant inconsistencies in their outcomes. For example, a study of three different ranking systems (PsyberGuide, ORCHA, and MindTools.io) demonstrated a lack of correspondence in evaluating top-rated Apps, indicating weak reliability (6).

Mhealth Apps present opportunities to improve access to high-quality mental health care. However, there is only limited evidence for their effectiveness, side effects, and cost-effectiveness (25). Therefore, as the numbers of Mhealth Apps grow, so does the need to regulate the field so that App users and referring clinicians can have sufficient information to choose an App. Therefore, we conducted a qualitative study to explore stakeholders' views and opinions and their understanding of “how to choose a Mhealth App.” Our stakeholders included an individual with lived experience of a mental health illness, two an AET developers, two physicians, and an App developer. The themes that emerged from the qualitative interviews were utilized to develop a survey that identified themes on how App users choose Mhealth App.

## Aim

We aimed to understand stakeholders' opinions and experiences on how to choose a Mhealth App. The objectives were to explore the stakeholder's views and experiences of existing guidelines for choosing Mhealth Apps, their weaknesses and strengths, barriers in their implementation, and experiences when using the app, including their decision process and barriers in this space.

## Methodology

### Qualitative study design and setting

This qualitative study consisted mainly of semi-structured interviews with stakeholders. Semi-structured interviews with open-ended questions, prompts, and facilitatory statements are considered to be the most suitable techniques for this study. These types of interviews provide researchers more control over the topics discussed without limiting the range of responses to each question, as is the case in structured interviews or questionnaires that use closed-ended questions (26).

### Sample recruitment

Key stakeholders were purposely recruited for their knowledge and experience developing AETs or Mhealth Apps and their experience using Mhealth Apps. Clinicians who have considered using Mhealth Apps in their practice were also consulted. Stakeholders were selected based on advice from experts in the field and through our network. The rationale was employing the maximum variation strategy, a convenience sampling method that maximizes sample heterogeneity to capture a breadth of views and perspectives (27). We first made a list of likely participants, who were then were contacted via email invitation and followed up by telephone. Those who consented were invited to an interview.

### Development of semi-structured interviews

We initially developed a list of areas that needed exploration through a brainstorming process. It was finalized in a group meeting conducted through Cisco WebEx. In addition to open-ended questions, prompts were agreed on to explore further areas of interest. It was considered essential that the participant's views be understood in the context of their background, so additional questions were added for each group of stakeholders. Interviews were conducted by members of the research team (CT and WK) who had prior experience with qualitative interviews. FN provided supervision throughout this process.

### Data collection

Participants were informed of the procedures and their rights to withdraw from the interview process at any time. Interviews lasting 45 min were conducted virtually using Cisco WebEx and recorded with the participant's consent. Participants were informed of privacy, and related risks before the interview and that identifiable information would be removed from the data except for a broad description of their background, such as “App developer”, that would be included in the analysis and results. Interviews were conducted between January 11th and 29th, 2021.

Each interview was fully transcribed and checked for accuracy. Transcription was started shortly following the completion of each interview. Participants were contacted if a response needed further clarification. Access to data was limited to the research team. The interview transcripts were returned to participants for comments, verification, and clarity concerning queries that arose from the analysis stage.

### Data analysis

Data were analyzed using thematic content analysis (28). This rationale was most appropriate to explore patterns across qualitative data. Each researcher (FN, CT & WK) analyzed the interview data multiple times to identify emerging themes and categories. We followed the principle of “emergent design” (27) when the respondent raised the issues that required further exploration. These issues were then tested appropriately in subsequent interviews with the participants. We also contacted participants by telephone to clarify areas of uncertainty when the data was analyzed.

Each interviewee was assigned a number for transcription and reporting. The data was primarily descriptive, with most themes emerging in response views. Two team members coded data separately to improve the reliability of the analysis. Finally, the data was reorganized into broader themes (e.g., views and opinions on Mhealth Apps) and categories (e.g., how Apps are developed and chosen). The authors held regular meetings throughout data analysis, facilitating the further exploration of participants' responses, discussion of deviant cases, and agreement on recurring themes. Two authors (CT and WK) independently analyzed the data using a thematic approach. When consensus was not achieved, FN helped reach an agreement.

Quotes are presented according to theme across multiple interviewees to highlight consistency amongst stakeholders and present contrasting viewpoints where applicable. Despite using the term AETs throughout this document, in the earlier stage of this project, the term “evaluative framework” was used in place of AET. As such, it is synonymous with AET in the quotes from key stakeholders.

### Study participants

Two interviewers (CT & WK) conducted six interviews with key stakeholders. Stakeholders who took part in our interviews adhere to five broad categories: App Developer (AD) from IT background with 5 years' experience, Physician (P) with no experience in AET or App development or evaluation, Physician Educator (PE) who was involved in development and evaluation of one mental health app in the past, AET Developer (AED) who was from IT background with 15 years' experience, and a Person Living with a Mental health condition (PLM) who had used a mental health app in the past. This person had a diagnosis of bipolar affective disorder. Two AET developers were interviewed to understand their experiences and opinions better. The App developer worked with a hospital. Participant's age ranged between 32 and 48. Both physicians were psychiatrists.

### Online survey study design and setting

#### Sample recruitment

The rationale of this anonymous online survey is to confirm the findings of the qualitative studies in a larger sample size. People were asked to participate if they currently used, or had previously used, apps. The survey was available for 5 days, from February 17 2021 to February 21, 2021. At the time the study was being conducted, those with lived or current experiences of mental health disorders and illnesses were neither actively sought out nor excluded.

To reach a wide range of participants who are likely to use technology, the survey was promoted on social networks (including Twitter, LinkedIn, and Facebook). We utilized a snowball sampling strategy, requesting retweets' and shares' from both participants and non-participants. Additionally, the original “tweet” on Twitter was' retweeted' on a daily basis. No incentives were offered.

#### Development of the online survey

The survey consisted of 12 questions. The topics that emerged from the qualitative study covered in the current paper served as the basis for the survey questions, which were prepared collaboratively by the research team. We aimed to obtain a broad picture of how end-users choose Mhealth Apps, which is an important issue for many AETs.

The survey consisted of 12 questions in total. The survey questions were jointly developed by the research team and were based on themes emerging from the qualitative study discussed in the current paper. Questions one through five captured demographic variables of interest, questions six and seven enquired about their past use (and rationale) of apps, questions eight and nine enquired about their existing process for choosing an app, while questions ten through twelve enquired about (a) who they would trust (e.g., hospital, government, clinician, IT app developer, etc.) to develop an app they would use; (b) what factors influence their app selection process, and (c) who they would trust to provide guidelines, or a tool, to choose an app. No personal details were collected.

#### Data analysis

We used SPSS v27 to analyze data. Descriptive statistics was use to describe data. Where the data were nonparametric, we used the Chi-square test to look at the frequencies of responses.

## Results

We identified six broad themes: (a) needs a opportunities; (b) views on MHealth Apps; (c) views and opinions on Mhealth App AETs; (d) implementation barriers; (e) system of evaluation; (f) future directions. Here we describe these themes and the categories under each theme.

### Needs and opportunities

#### Mental health apps

With smartphone and web technology expansion, Mhealth Apps have exponentially increased in popularity and usage among service users, health service providers, and researchers in the past 10 years. Comments by our stakeholders reflected the potential of this technology to expand service delivery, improve clinical integration, and for the potential to further individualize the App experience for users with the frequently improving technology.

One participant reflected on increasing demand for advice, “*Well, people do come and ask, what are the resources [Mhealth Apps]? Because it's an issue [lack of resources], you do wish that you had more resources we could recommend to people.” (P)* Another participant highlighted the potential for improving access to psychotherapies through Mhealth Apps, “*One of the challenges, of course, is with someone who may not have the resources, may live in an area there's not a lot of clinicians, being able to access CBT might be difficult” (PE)*.

Although not directly referenced in most interviews, the backdrop of these interviews is amongst the COVID-19 pandemic in which the context of comments surrounding the Mhealth App comes amid stay-at-home orders, challenges in face-to-face contact with service providers, and the rise of telehealth. Most interviewees highlighted the potential for mental health apps to present a unique option to integrate with traditional services and provide psychoeducational or therapeutic material when other traditional supports are not available.

“*I believe there's more urgency now to develop Apps” (P)*, another stakeholder said, “*...especially amidst the [COVID-19] pandemic in a time where a lot of services are not readily accessible, and travel is quite challenging. So, for me, I see a lot of promise” (PE)*.

#### AETs (Assessment and Evaluation Tools)

According to AET developers, concerning the rapid evolution of technology and expansion of the Mhealth App space, there seems to be a growing need for oversight in the form of formal AETs. We must move away from the user's reliance on the commercial App Store's rating and review evaluation system. One AET developer said:

“*If they're intended for therapeutic use, it really needs a higher level of scrutiny because people who need treatment need to get treatment on evidence-based approaches. You wouldn't walk into a cancer clinic and expect to get a treatment that hadn't been approved by anyone, so why would you (not do that) in the digital space if you need treatment for mental health?” (AED2)*.

This same AET developer also expressed concerns about the existing App evaluation systems that might lead to the use of Apps that might not offer what they claim.

“*The App user may have wasted time thinking that something was therapeutic. So, the idea that we can use crowdsourcing, because people we don't even know who rate it, that gave it five stars, who weren't scientifically trained or medical professionals to evaluate it, those kinds of websites and frameworks are showing up everywhere, and I think it may mislead people on really that is an effective App that has therapeutic benefits vs. that don't, and that may delay treatments” (AED2)*.

Another AET developer further explained that established AETs could help individuals navigate the obscured motivations of App developers in a quickly changing and profit-motivated field.

“*It's probably the market forces that are determining [the variety and quality of apps]. It's an economic matter. There's not much input from family members, clinicians, patients, even payers. So, it's just the free market that has shown us what a market will support” (AED1)*.

### Views on mental health apps

#### App development process

The interviews with our key stakeholders made it apparent that there is an increasing gap between how Apps are currently being developed and their views on how they should be developed to maximize accessibility and effectiveness for users. The primary examples raised were the lack of input from various stakeholders, functionality, language, and App looks.

The App Developer acknowledged the lack of stakeholder engagement, “*I think it's just getting people involved, and I know it's difficult [for some App developers] to find the right people. Especially if someone isn't in the mental health environment.” (AD)* The need to include stakeholders in the App development process was also emphasized by the physician educator, “*[it's so important] the co-development and review or design with a clinician, with expertise in [App evaluation]” (PE)*.

Both the App developer and the person who has lived experience of a mental health illness emphasized the need for appropriate use of language in terms of an App's ease of use and its cultural aspects.

The App Developer stressed the importance of language, “*So, [in some languages] the word for like say schizophrenia or mental health might not be there per se. So, you have to make sure it's closely relevant and stuff like that.” (AD)* This was further emphasized by the person who has lived experience of a mental health illness, “*The other thing is the language that's being used. Like, how do they welcome me? How do they talk about mental illness?” (PLM)*.

They also spoke about the aspects of the App that matter to them a lot.

The person who has lived experience of a mental health illness said*, “How it looks [comes first], then functionality. Is it easy for me to find certain things? Those things are important to the experience, the user in my eyes.” (PLM)* The App developer raised similar issues*, “...for instance, making sure color contrasts are at a good level, a font size [that helps with] accessibility for websites and Apps” (AD)*.

#### How apps are chosen

Among stakeholders, the primary App users were the person who has lived experience of a mental health illness, for whom most Mhealth Apps are targeted, the App developer (as a Mhealth App user), and the clinicians (in this case, psychiatrists) who are possibly advisors assessing and recommending Apps for use in their clinical practice. Each of these stakeholders stated that user experience, credibility, or validity (i.e., the App is providing what it says it's providing) is of utmost importance. Credibility also extends to the App developer themselves. Do they have a track record of quality Apps, positive feedback, and developed relationships and collaboration with health providers?

Our stakeholder interview with a person with lived experience of a mental health illness was illuminating, specifically the conversation about selecting a Mhealth App. In terms of how they chose the Apps and their initial impressions, they said:

“*If I'm on the [Google] Play Store, and I'm looking up Mental Health App, or Mental Health Tracker. It's kind of a—which one is the prettiest? Which one is the most eye-catching or that I think is easy to use? That might not be the best one for me, but it's easy to use. At the time when I'm depressed and anxious and all the other stuff I'm dealing with. I just want it to be easy” (PLM)*.

They further explained what features they liked the most in an App they used,

“*Daily reminders, motivating, keeping you accountable like pill tracking and habit tracking put all into one is very helpful. Then looking, engaging, and having fun can be beneficial. Separating it from the medical-looking App is cool.” (PLM)* They liked the idea of an animated character, “*I like the idea of having this buddy” (PLM)*.

They shared their reasons for trusting the App. They also emphasized the importance of cultural and age-specific issues.

“*There was mention of it coming from [health services in a Canadian province], so I really did like that it was Canadian mental health. It came from that industry. My experience with it is that what makes this one so different from the ones I've seen is that because it sounds like it's made for younger folks, it's so, I don't want to use the word simple, but it's not as medical” (PLM)*.

Finally, they explained the reason for their engagement with the App, “*There's a check-in question every day, and then there are quests you can do, and you can gain points. Frankly, I don't think they go anywhere, but the idea of collecting points really entertained me at the time, and it just made me feel like I can achieve things. What's also great is that there's this motivational messaging that's around it” (PLM)*.

On the other hand, the AET developers shared their concern that it may be that Mhealth App users are not carefully selecting an App that best suits them, and this is reflected in recent research showing most Apps are deleted within a week of download (14). One AET developer said:

“*The reality is that most Apps aren't used beyond the first 2 weeks. Over 95% of them aren't used beyond 2 weeks, and there have been formal studies to show that.” (AED2)*. This point was also emphasized by the second AET developer who said*, “I think the usability, [one paper] in 2019 shows you 90% of people stop using the App in 10 days. So, the engagement crisis is pretty high” (AED1)*.

#### How apps are chosen- online survey

Two-thirds (67%) of the one hundred and seven survey respondents indicated they have previously used an Mhealth App. Of app users, 37% had used the Mhealth App for a specific mental health problem, and 33% of respondents had used a mHealth App for general wellbeing. The remaining respondents used a Mhealth App for research, did not know, or preferred not to share their reason.

*In response to the question, “Where do you look to help you choose a mental health app?” most respondents (66%) stated that “app store ratings and reviews” were their source of primary consideration. In terms of important factors that individuals look for in a Mhealth App, the most common response from respondents was “functionality” (83%), followed by “ease of use” (77%), “cost” (69%), and “research evidence” (57%). When responding to the question, who will you trust to produce a mental health app, the most common answer was “a hospital” (68%), followed by “mental health clinician” (62%) and “a government agency” (59%)*.

*The survey respondents” overwhelming choice in response to their preferred source for advice in choosing an app was a “health professional” (84%). Finally, when asked who is best suited to develop guidelines for choosing a mental health app, the most common answer was “health professionals' (70%), followed by “university researchers” (59%) and “individuals with lived experience” (43%). Please see* *Appendix B* *Figure 1 for participant demographics and* *Appendix B* *Figure 2 for details of the last five questions from the survey*.

#### Concerns around Mhealth apps

Several concerns were raised by the stakeholders, specifically around privacy, data collection, large amounts of text, and issues related to digital equity.

For example, the person with lived experience of a mental health illness said, “*The thing I didn't love [about the App], was there were a lot of questions, in the beginning, it's a bit of a barrier. For me, it was reading text. It was just a lot of text*.” (PLM). They were less concerned about privacy and security, “*I didn't have to create an account [what they liked about the App], which is great. Like I don't want to put in my email, I don't want to put my full name and that kind of stuff. I want to see if this is something that I want to invest my time in before I create a full account and tell you all my information” (PLM)*. They further explained their point by saying, “*One thing that always comes up is privacy and where that information goes” (PLM)*. Other participants reiterated this last point about privacy, “*I think privacy is the main issue always.” (AD), and “I think one of the issues would be privacy” (P). One* physician further discussed the risks and harm as they said, “*Is there a fallout to delivering therapy through this App?” (PE)*.

Nearly all the participants discussed the lack of evidence for most Apps and how evidence should be built around the Apps.

“*Well, I think the work needs to be done, to do some sort of a clinical trial of its effectiveness” (P)*. Another participant said, “*I think you never know (for other Apps) the involvement of people if it's evidence-based, research-based” (AD). One* AET developer said, “*The App needs to be evidence-based as an App can be, at least designed by experts having face validity.” (AED1)*.

Finally, one of the physicians who use Apps in their practice discussed how important it is to consider “digital literacy” and make sure the person advised of using an App has the necessary technology available. They said:

“*I usually do ask about connectivity and access to Wi-Fi. What kind of devices they have, and [their] familiarity with using devices, if I am recommending Apps as a potential resource. Similarly, if I were asking someone to utilize videos for education or mindfulness or relaxation on YouTube, I would ask about their familiarity with YouTube” (PE)*.

#### Mhealth apps in clinical practice

Without standardized guidelines, a physician's familiarity with technology often correlates with how they incorporate Mhealth Apps into their practice.

One physician said, “*I haven't used any [Mhealth Apps]. I have heard of some recommendations going back and forth between clinicians around the Apps, but specifically, I have not recommended any” (P)*. The other physician, on the other hand, who developed Apps in the past, said, “*I can definitely say I've had some patients who are able to use [Mhealth Apps] and integrate them in their day-to-day and then report back as part of our continued follow-up care” (PE)*.

There was a discussion with physicians about the potential for Mhealth Apps to be another therapeutic option for them to use when working with a patient. In addition, the physicians discussed issues related to the appropriateness of when to recommend Apps.

One physician said, “*I do see a number of young people too now, and they're probably more willing to try Apps than others” (P)*. Another physician said, “*There are opportunities to be one part of the toolkit for a clinician, whether it's an in-person type of care and augmenting it or it was being completely virtual. So, I do see that it holds promise in terms of decreasing barriers for waitlists and increasing access” (PE)*. This physician also expressed some concerns over the App use, “*The other piece is I do have a worry about is that sometimes we'll get to a point where Apps are thought to be the replacement for other types of interventions” (PE)*. The physician mentioned the use of Apps within a stepped care model of care “*So, let's say if the access to actual therapy is a bit delayed or there is a longer waitlist, it's probably better than nothing to start with an App” (P)*.

Finally, in terms of improving Mhealth App use in clinical practice, participants offered helpful advice:

“*There are a lot of wellness Apps out there, for example, and [we should] make sure that the clinicians know how and when to use them in the treatment algorithm or pathway” (PE). One* of the AET developers said, “*I think patients are comfortable telling us they're using Apps, but we certainly make it part of our business.” (AED1)*.

#### The usefulness of the Mhealth apps

All the participants agreed on the usefulness of the Apps, albeit with some reservations. One participant said, “*I found it very effective to the point where I actually recommend this App to other people” (PLM)*. Another participant said, “*I've seen transient benefits that really drop off overtime where the first couple of weeks people are engaged, and then after a while, people have ignored the alerts to log in or to participate” (PE). One* AET developer said, “*I think that as self-help tools, the evidence from the peer-reviewed literature is that they're pretty limited [in usefulness]. They don't harm you, but it's a pretty small effect when used in the context of a clinical relationship” (AED2)*. The other AET developer expressed their concerns as “*it's not the problem that people don't want to use something. It's just, what's out there isn't actually what they need or want, or just isn't useful” (AED1)*.

### Views and opinions on AETs for Mhealth apps

#### Need for AETs

As discussed, the need for AETs in a fast-paced and lucrative field of mental health provision is essential to help guide users amongst the numerous options, highlight safety, and privacy concerns, and provide the necessary tools for informed decision-making.

One physician said*, “I think a framework is critical because a checklist would be ideal for clinicians.” (PE) One* AET developer emphasized the need for developing AETs by saying*, “It's an important decision of what you're using, and there are risks and benefits. It's different from downloading Candy Crush, even though they're marketed the same, and sometimes look the same.” (AED1)* The person with lived experience of a mental health illness said*, “If there are these resources like a list of Apps, I want to know so I can share them with other people” (PLM)*. Similarly, another AET developer said, “*Well, no one's been hurt. Well, we don't know that. You may have delayed treatments because of that [if the App was not effective]” (AED2)*. This same AET developer also emphasized the need for better policies, “*we'll need to have better policy oversight for that [better regulation of Mhealth Apps]” (AED2)*.

#### Development of AETs

The AET developers provided insight into how the AET should be developed. One AET developer discussed their AET development process in some detail.

“*We had several iterations of the framework. We presented it at different meetings for several years. We worked with it, with our different patients in our clinics, and we got a feel for what pieces mattered to people and which didn't. Then eventually, we went to the research literature and looked at other frameworks. We looked up all of the research literature, and a little bit of the gray literature around it” (AED1)*.

Key considerations included whether the AET has been subjected to evidence-based research, its development was carefully documented and included in published reports, and whether there was stakeholder involvement (and to what degree).

“*I think that should just be a group of people from all these different backgrounds. You'll have potential developers involved as well, but researchers, clients, subject matter experts, anybody really” (AD)*.

“*So, we were at local clubhouses in the community” (AED1)*.

“*We are organizing panels of experts. We assembled people who were both scientists, and we consulted people with lived experience as well”* (AED2).

#### Awareness and use of AETs

Apart from the AET developers and the physician with experience in App use, stakeholders (including the App developer) either reported that they were not aware of the existence of AETs for Mhealth Apps or, if they had, they expressed concern that the AETs largely go unknown to the average App user. Thus, awareness of AETs appears to be a key component for widespread implementation.

“*No [not aware of an AET]. There's no framework that we're checking off that we've been following per se” (AD). One* physician emphasized the need for Governmental agencies to be involved, “*So, I think there needs to be more collaborative work around it, and possibly the government should play a bigger role in this because they should assume some responsibility in the delivery of care” (P)*. The person with lived experience of a mental health illness at first reported no knowledge of AETs upon further inquiry, said*, “If I'm in a place where I want to look for an App and the guideline is super easy, like low engagement, low reading, then yeah, I might be interested to see what are some points that have to be within an App. Or for there to be sort of like a flag at the beginning. Even within the description, it says that this meets that guideline. Then I'm like,' Great,' I would assume this was good” (PLM)*.

#### Areas for improvement

All interviewees reported that a potential weakness of the AETs was their exclusion of criteria that report on cultural issues and features that personalize the App experience to a user's identity. Most AETs in our review did not include culture, language, ethnicity and race, lifespan, and gender considerations in their selection criteria. One AET developer highlighted the importance of age and language consideration as:

“*The vast, vast, vast majority of Apps do not ask your age and consent of your parents if they're collecting clinical grade data. And that's a legal problem because those Apps are collecting things with that. So, you couldn't do that with a website. So, there are rules for that, so we made special notice of how you collect data for youth and the consent process. But other than that, all of the other criteria are age-independent” (AED2)*.

Similarly, it was highlighted how vital disclosure of conflict of interest and financial disclosure by AET developers is to ensure that AETs are not promoting one App or another:

“*[An AET] needs to be explicit on everyone's in terms of conflicts of interest, right? Because people who may be recommending Apps, especially if they were developers of the App- Or have a financial interest around the advisory boards and things like that. Not everyone discloses their conflicts of interest*” (AED2).

Finally, it was pointed out that most AETs were not developed using rigorous methodology. As a result, none of the AETs have been formally evaluated. Similarly, most AETs do not offer advice on how to use their selection or evaluation criteria:

“*The framework [development process] is not rigorous. It has to be measurable, and it has to be measurable in a reliable way. You can't just use any old survey of questions. You should be using something that's been validated” (AED2)*.

### Implementation barriers

#### Mental health apps

Despite the promise that Mhealth App technology holds, numerous stakeholders expressed concern over the barriers to users before widespread implementation is possible. These barriers include inequities in access to technology (i.e., the digital divide), skills to utilize technology (i.e., digital literacy) thoroughly, and cost barriers.

Like this participant said, “*So, technology readiness is a problem, and training and support” (AED2)*. One AET developer discussed the need to improve digital literacy, “*I think the best way really to link digital literacy with App evaluation is that they kind of go together, if you're looking to improve mental healthcare” (AED1). One* of the physicians said, “*I do think that digital equity is an important piece about how we ensure that Apps are culturally adapted, responsive, and tailored for specific patient populations? (PE)”*. They further emphasized the need for equity in this area*, “It's unrealistic to think that all Apps will be able to be designed for all populations. We did this in one of our libraries, our App libraries, to flag whether there were Apps tailored for indigenous or black patient populations given the need for cultural sensitivity and being adaptable for those patient populations. There were not many (Apps), as you would imagine” (PE)*.

One AET developer offered insights on implementing the AETs in clinical work and suggested possible changes in health systems to improve the use of Mhealth Apps.

“*So social workers usually are asked to do multiple things, but now we can add them to be clinical support, digital interventions, right? And so, I think we need to look at roles and responsibilities, and who needs to be involved in the technical aspect, and what training and support we give to them. So, I think a lot of people are overwhelmed, and the integration can't happen without some planning and additional support” (AED2)*.

The person with lived experience of a mental health illness expressed their concern regarding the cost of the Apps and their views regarding the authenticity of the App builder.

“*First of all, it's just a financial barrier. I wouldn't want that. Then I would think about yeah, it is a bigger privatized business kind of thing. I would like it to come from a mental health commission or a hospital. I wouldn't want it to come from a company. I mean, those are companies, but you know what I mean” (PLM)*.

The physician educator described their concerns regarding privacy, security, and risk awareness as a potential barrier to implementation:

“*So, I think more and more we recognize the importance of digital safety and privacy, right? That's a huge key. I know there's lots of research coming out about how a lot of these Apps do not disclose their privacy regulations and processes explicitly, either in the App itself or on the website you're downloading from. It's so important to inform patients about that. So, I do think people using their information secondarily for other sources is a risk and also might be a deterrent for some clinicians, including myself” (PE)*.

#### The AETs for mental health apps

There was full agreement amongst our participants about the necessity for AETs to assess quality for Apps, but numerous barriers were highlighted about the widespread adoption of AETs. Interviewees either were not aware that AETs existed, or individuals with experience in the field (i.e., AET developers) expressed their concern that the AETs were not visible or widely disseminated by the individuals who most need it.

“*One, to do it properly [implement a framework], you need time and train people on how to do that. And the question is, where do we, [or] how do we find the money to help train people to do that? If you're not trained in how to do these assessments properly, you're not going to get a consistent or reliable result” (AED2)*. Another participant emphasized the need to improve digital literacy to facilitate the implementation of the AETs, “*So, I think the best way really to link digital literacy with App evaluation is that they go together if you're looking to improve mental healthcare” (PE)*.

It was also discussed that the language used, considerations implemented from a user's perspective, and the format in which the AET is disseminated were significant factors that affected the degree to which Users or clinicians could widely adopt AETs.

“*But if I'm in a place where I just want help, and just like a quick response to find something, ideally it works, and let's just go with that. I probably wouldn't do this [review an AET]. I probably wouldn't take the time to do the research” (PLM)*.

### Difficulties in the evaluation of Mhealth apps

Numerous stakeholders focused on the deficiencies of the present research on Mhealth App effectiveness, raising questions of the ability to control variables of interest in long-term studies conducted outside of the research lab and the competing interests of marketing and scientific motives. Of particular interest were the comments that raised concerns about the rigor and design of studies that tout the Mhealth Apps' effectiveness. For example, results are often reported from small and quickly completed feasibility studies instead of large-scale randomized controlled trials (though there are difficulties in conducting these).

“*It seems like a piecemeal approach. The clinicians are on one side, and the App developers are on the other side. They seem to have a different focus [on what the user wants]” (P). “Also, a lot of them [evaluations] have small sample sizes. We found that there are many Apps with limited evaluation, so there's a lot of methodology problems with it. If the App is used in conjunction with face-to-face services, you need to know to what extent the face-to-face services contributed to the outcome vs. the App” (AED2)*.

Some of the participants highlighted the lack of an agreed definition for the effectiveness of the Apps.

“*People define ‘effectiveness' vastly different, or they don't define it at all, which makes it even more challenging to decide what they're recommending. I think problem number one is that there isn't an agreement on what is considered to be effective” (AED2)*.

#### Evaluation of AETs (problems)

As mentioned earlier, none of the AETs have been evaluated. One of the interviewees with extensive experience in assessing AETs highlighted the potential for the risk involved in using AETs that do not have a consistent and rigorous methodology in their design, implementation, and testing. A set of guidelines for the evaluation of AETs and aid in the development of AETs specific to the health field is a goal.

“*I think there are a plethora of frameworks that aren't scientifically based, and that causes a huge problem because some of them can give an inconsistent level of rating that makes it look like an App works when it hasn't been scientifically evaluated for effectiveness” (AE2)*.

### Future directions

Key informants agreed that the field was still in its infancy, with much work to be done (as expected with the technology's novelty). This included the desire for more rigorous development of AETs and a broad re-conceptualization of critical factors to be evaluated and how they should be presented to users and clinicians in an accessible (and innovative) way and who should be involved in that process.

“*I think there's a lot of room for improvement [in the development and evaluation of Mhealth Apps]” (P). One* AET developer said, “I mean, it's a strange evolving space” (AED1).

Further, the clear identification of competing interests of those embarking on App evaluation, and the development of AETs, was of the utmost importance to all key informants. There was agreement that the distinction between for-profit and non-profit was essential in this domain. The need to be transparent and clear to App users and health providers. It may require further steps of regulation.

“*So not only do we need to use [an AET], but we need to use one that is evidence-based, scientifically-based, that's transparently evaluated without conflicts of interest, and there has to be oversight of how we're implementing the framework, and then how people are actually using the Apps, and based on those evaluations, circle back to, maybe we need to recommend other Apps, or maybe we need to implement them differently” (AED2)*. Or, as the other AET developer said, “*I mean, eventually there's going to have to be enforceable standards for these” (AED1)*.

Special attention could be paid to the AET developers and researchers who have spent a lot of time considering development goals and stress-testing the AET's implementation and effectiveness. Their recommendation of how the field can ideally advance is illuminating.

“*So, I think we need some advocacy by groups... and we need transparency in science. [Because we have] a vaccine that gets put in our arm that has been transparently and scientifically evaluated. And so, if we expect that of a vaccine if we expect that of a drug, we should expect that with [these] interventions too” (AED2*).

## Discussion

As far as we know, this is the first qualitative study to explore stakeholders' views and opinions on existing AETs. The overall purpose of the AET is to guide end-users on how to choose a Mhealth App. App developers, AET developers, and clinicians can have different ideas on selecting Apps. Overall, many stakeholders, including people who use Mhealth Apps, are unaware of AETs for Mhealth apps. Even though consumers consider user ratings when selecting an App, they do not portray an accurate image of App suitability, assuming they are not reinforced by empirical evidence (8). Thus, another way for consumers and clinicians to choose an App is to use an App review platform (8).

According to Schueller and Wykes (29), four principles should be used when the consumer selects an application to download called the Transparency for Trust (T4T) principles. These include privacy and data security, development characteristics, feasibility data, and benefits (29). It should be the responsibility of the App store to provide the information from these principles for a health App (29). The majority of respondents (66%) to the online survey stated that ratings and reviews on the App Store and Play Store are the main factors they take into account when choosing an app.

Participants in our study emphasized the need for equity, diversity, and inclusion for the AETs. Most participants emphasized the need for further improvements to Mhealth Apps to address areas of concern such as cultural sensitivity. Both Apps and AETs appear to pay little consideration to cultural, gender, language, and lifespan issues. Since the COVID-19 pandemic and mainstream recognition of systemic racism, greater attention is being paid to digital health and its capabilities in increasing access to and standards of behavioral health care (30). Regardless, this greater dependency on digital health during the pandemic has continued to create more considerable health disparities in racial and ethnic minority groups, in turn amplifying the digital divide. This is an issue for racial and ethnic minority groups that already suffer health disparities and a greater reliance on digital health technologies, increasing the digital divide (30). In terms of AETs, Zelmer et al. (31), highlight that organizations or individuals recommend Apps based on various factors; however, it is uncertain if these factors are generalizable to different groups based on culture, gender, and language (31). According to leaders within the Computing Community Consortium and Society for Behavioral Medicine from multidisciplinary fields, they have agreed that reducing inequality would mean that these minority groups are involved “at all stages of intervention design, implementation, and evaluation” (32).

Numerous stakeholders, including a person with lived experience of a mental health illness, were concerned about privacy and safety concerns around App privacy and invasive data collection. This is a concern because most health Apps gather a lot of information on personal data, which affects health, so it is imperative to ensure safety, validity, reliability, privacy, and security. The Organization for Economic Co-operation and Development (OECD) developed a report in 2017 that stated the use of poor quality and non-medical health Apps brings about many ethical, legal, and governance issues. Thus, there needs to be an international agreement on basic standards for quality assurance and an easy source for App developers and users to follow (33). Analysis of mHealth Apps privacy is usually based on the user interface, communications privacy, and the privacy policy. The guidelines for assessment in existing AETs are less objective and heterogeneous, particularly for user interface and privacy policies. Hence, a more comprehensive evaluation needs to be created for these policies to produce more accurate results after using these assessments (34). Torous et al. (35), suggest the following strategies to improve data safety and privacy: data use, storage and sharing policies that are transparent to users of the App, standards agreed upon for data usage, storage and sharing, end-user awareness of data being shared with external partners, and the end-user should be given the option to stop sharing their information (35).

The use of Mhealth Apps in clinical practice varies among clinicians depending on their background and interest. Interviewed physicians described problems related to access to technology and digital literacy. Similarly, functionality (83%), and ease of use (77%), were the two biggest considerations that online survey respondents indicated they looked for in an Mhealth App. According to Nouri et al. (36), populations with limited digital literacy are less likely to use mobile Apps. Many of these groups are vulnerable or minority populations, that regardless of having smartphones, still struggle with text- messaging (36). Therefore having a phone and using it daily does not mean someone is able to use basic smartphone functions use such as texting. Digital health literacy is the skill to evaluate health information from varying electronic sources and use that information toward resolving a health-related problem (37). Accordingly, prior to mHealths' most significant hurdle is digital health literacy and requires a combination of both general and health literacy (37). In the paper by Smith and Magnani (37), a Digital Universal precautions for eHealth literature health care organization was generated to improve the accessibility of eHealth services for everyone (37). The safeguards included: creating an interdisciplinary team including programmers, designers, and patients, creating user-friendly and convenient digital media resources that are actionable and evidence-based, the delivery of media through video or audio to increase communication for those with limited literacy, and interactive services where patients can tailor information to their needs. Although, in the case when individuals do not have access to the internet, organizations should supply devices or the means to the technology (37).

The existing research methods may not fully consider complexities in evaluations of Mhealth Apps. Engagement with Mhealth Apps is a significant issue. The lifespan of most Apps is very short. Furthermore, there are many mhealth Apps available to consumers. However, only a few meet the requirements to be incorporated into a healthcare system. Considering App development and change occurs very quickly, the complicated and prolonged clinical studies that determined the effectiveness of just one App cannot keep up with the ever-changing App market (38).

Moreover, there is insufficient agreement on the minimum methodological guidelines that should be used for App evaluation which has caused a more intensive evaluation that includes mHealth randomized controlled trials (RCTs) being performed in countries (39). Technology is fast-paced, and an App platform being evaluated using RCT can become outdated during the clinical trial (39). Other factors that affect App evaluation include incorporating an interdisciplinary team, elaborate sociotechnical aspects that the mHealth success relies on, and external factors such as financial, human, and time resources to perform these evaluations (39).

While several AETs are available for end-users, not many of our stakeholders were aware of AETs. Implementation of an AET appears to be a significant barrier. The stakeholders emphasized the need for advocacy and engagement of health professionals in the implementation process for AETs. This is evident in the online survey, where 68% of participants said they would trust a hospital the most to create a mental health app. 84% of respondents would prefer a health expert to provide guidance on Mhealth Apps, and 70% would like health care providers to design an AET. Aside from health professionals, individuals outside the medical field have not had the chance to voice their opinions on these assessments of health aids even though they know the benefits of mHealth. Along with technology, developers investigating the possibility of “ever-present self-management systems,” should have a mutually beneficial relationship with the medical system, so evaluation frameworks are safer for end-users, transparent, and trustworthy. These findings are very similar to those in Van Daele (40), which highlights all stakeholders involvement is a necessity. As such, health authorities, patients, and mHealth developers need to be actively involved during the process of creating evaluations for mHealth (41). In general, all stakeholders involvement is a necessity to disseminate superior health care and digitally delivering mental health (40).

### Limitations and future directions

The generalizability of the findings from this study may be limited due to having a limited number of participants and individuals with lived or living experiences of mental health illness and a small sample size overall for the online survey. In relation, there were considerable time and financial restrictions. We used convenient sampling that further poses limitations on generalizability of this study, for example no psychologists or nurses were included in this study. Another limitation was the general lack of agreement within the field surrounding terminology and definitions of assessment criteria that may have led to misinterpretations for qualitative purposes, even though expert opinion was sought.

More research is required to understand health Apps for physical and mental health, the buying tendencies of people who use Mhealth Apps, the perspectives of healthcare professionals, and the impact of digital literacy and access to technology. AETs cannot be more functional until more research is done on end users' requirements, perceptions, and expectations. Research funding streams specific to Mhealth Apps will be a wise investment to assess the quality of mental health care apps. Finally, understanding the perspectives of App developers and engaging them to regulate health Apps is critical.

## Conclusion

This qualitative study explored stakeholders' views of AETs (Assessment and Evaluation Tools) for Mental health Applications (Mhealth Apps). Sparse information or advice is available from authentic sources on “how to choose a Mhealth App”. Many people are unaware of existing guidelines for choosing Apps, developed mainly by academics interested in this area. The problem is further compounded by the fact that very little evidence is available for the effectiveness of the Apps, and the existing methods in mental health research do not provide clear guidance on developing and testing Mhealth Apps. Stakeholders agreed that Apps could significantly impact mental health if evaluated adequately through a rigorous methodology and implemented effectively. The stakeholder commented on a need for clear evaluation guidelines for end-users need to be able to trust the reliability of App evaluations. The primary barrier described by the stakeholders was the *implementation* of apps in healthcare delivery services. There is a clear need for more research in this area.

We quantified some of the themes emerging from the literature review and the qualitative interviews using an online survey that mainly focused on the end-users. This survey confirmed that for Mhealth App users, the primary source of information remains app distributors such as Google's Play Store or Apple's App Store since the app users had limited knowledge of the AETs. The participants considered functionality, ease of use, and cost as the three primary reasons to choose an app and were less concerned about evidence from research, privacy and security (factors that AETs and the current regulations heavily rely on). The primary focus of Mhealth app users, and of Mhealth app developers, appears to be on the utility provided to the user. Finally, the app users considered health professionals to be their overwhelming choice to guide them in selecting an app. It is yet to be seen whether health care professionals and health care organizations around the world are ready to step to the forefront.

## Data availability statement

The datasets presented in this article are not readily available because the data belongs to The Centre for Addiction and Mental Health. Requests to access the datasets should be directed to farooq.naeem@camh.ca.

## Ethics statement

Ethical review and approval was not required for the study on human participants in accordance with the local legislation and institutional requirements. Written informed consent from the participants was not required to participate in this study in accordance with the national legislation and the institutional requirements.

## Author contributions

WK, CT, and FN were actively involved in conducting the semi-structured interviews for the study and in the completion of the analysis of the interviews. WK wrote the first draft of the manuscript with the assistance of FN. BJ wrote the second draft of the manuscript with the assistance of WK and FN. All authors reviewed and edited the manuscript and approved the final manuscript for submission to the journal.
