# Supplementary material for: Stakeholders' views and opinions on existing guidelines on “How to Choose Mental Health Apps”
Source: Front Public Health. 2023 Nov 22;11:1251050. doi: 10.3389/fpubh.2023.1251050 (PMC10703154; doi:10.3389/fpubh.2023.1251050)
Supplement: Supplementary file 1 [file Data_Sheet_1.docx]

# **Appendix A**

# **Glossary**

**App (Application):** An app is a software program that performs a specific function directly for an app user or, in some cases, for another application.

**Assessment and Evaluation Tool (AET):** A set of guidelines, or framework, used for the evaluation and assessment of a practice, intervention, concept, or parameter.

**Assessment tool:** An assessment tool assesses a concept or a parameter.

**Customization:** An app user manually makes changes to achieve their preferred experience.

**Device Software Function (DSF):** In a medical context, device software function refers to software that transforms a person’s input from the interactive interface of the device (e.g. keyboard, touchscreen) as either embedded software *within* a medical device, Software in a Medical Device (SiMD) (e.g. software inherent in a medical device, such as a CT Scanner), or as software that *is* the medical device, Software as a Medical Device (SaMD) (e.g. a medical assessment tool intended for use on a smartphone in the form of a mobile app).

**Digital divide/Digital equity:** The digital divide refers to the inequity of access to information and communication technologies (including health information and digital-based treatment options) between some communities and other communities. Barriers to digital information or communication technologies for individuals with lived or living experience of mental health problems or illness can reinforce health inequality.

**E-Mental Health:** An overarching term used to describe the application of information and communications technologies in the health sector. It encompasses a whole range of purposes from purely administrative through to health care delivery by Health Canada.

**Evaluation:** The testing of a practice or intervention**.**

**Framework:** A framework describes a possible outline of an area within which some work or practice is completed.

**Guidelines:** Guidelines describe best practices in an area usually based on current evidence.

**Health app:** An application program, either web-based or on a mobile platform, that offers health-related services.

**Mobile app (Mobile application):** A mobile application is a software application designed to run on a *mobile device.* Mobile applications often stand in contrast to *desktop applications* designed to run on desktop computers and *web applications* that run in *mobile web* *browsers* rather than directly on the mobile device.

**Mobile device:** A mobile device (or handheld computer) is a computer small enough to hold and operate in hands, such as a phone or a tablet.

**Mobile Health (MHealth):** Mobile health refers to the practice of medicine and public health supported by mobile devices such as mobile phones, tablets, personal digital assistants and wireless infrastructure

# **Appendix B**

*Figure 1. Online Survey demographics (n=107)*

|  | *Percentage* |
| --- | --- |
| ***Age*** |  |
| *19-30 years* | *32%* |
| *31-45 years* | *38%* |
| *46-60 years* | *24%* |
| ***Gender*** |  |
| *Female* | *60%* |
| *Male* | *33%* |
| *Agender, Non-Binary, Transgender and Other* | *6%* |
| ***Ethnicity*** |  |
| *White* | *61%* |
| *Asian* | *25%* |
| *Other ** | *5%* |
| ***Responses from Canada*** |  |
| *Yes* | *75%* |
| *No*** | *25%* |
| ***Stakeholders*** |  |
| *Health Care Professionals* | *43%* |
| *Lived or Living experience of a mental health problem or illness* | *40%* |
| *Others **** | *17%* |

**Others: Latin American, Middle Eastern, black African or Caribbean, one of mixed heritage.*

***No: United States of America, the United Kingdom, Pakistan, India, Japan, Germany, Brazil, and Bangladesh.*

****Others: researchers, app developers, students, caregivers or family members of persons with lived or living experience or respondents answered, ‘I don’t know’.*

**—**

### ***Figure 2: Results of the Online Survey (Questions 8-12)***

**
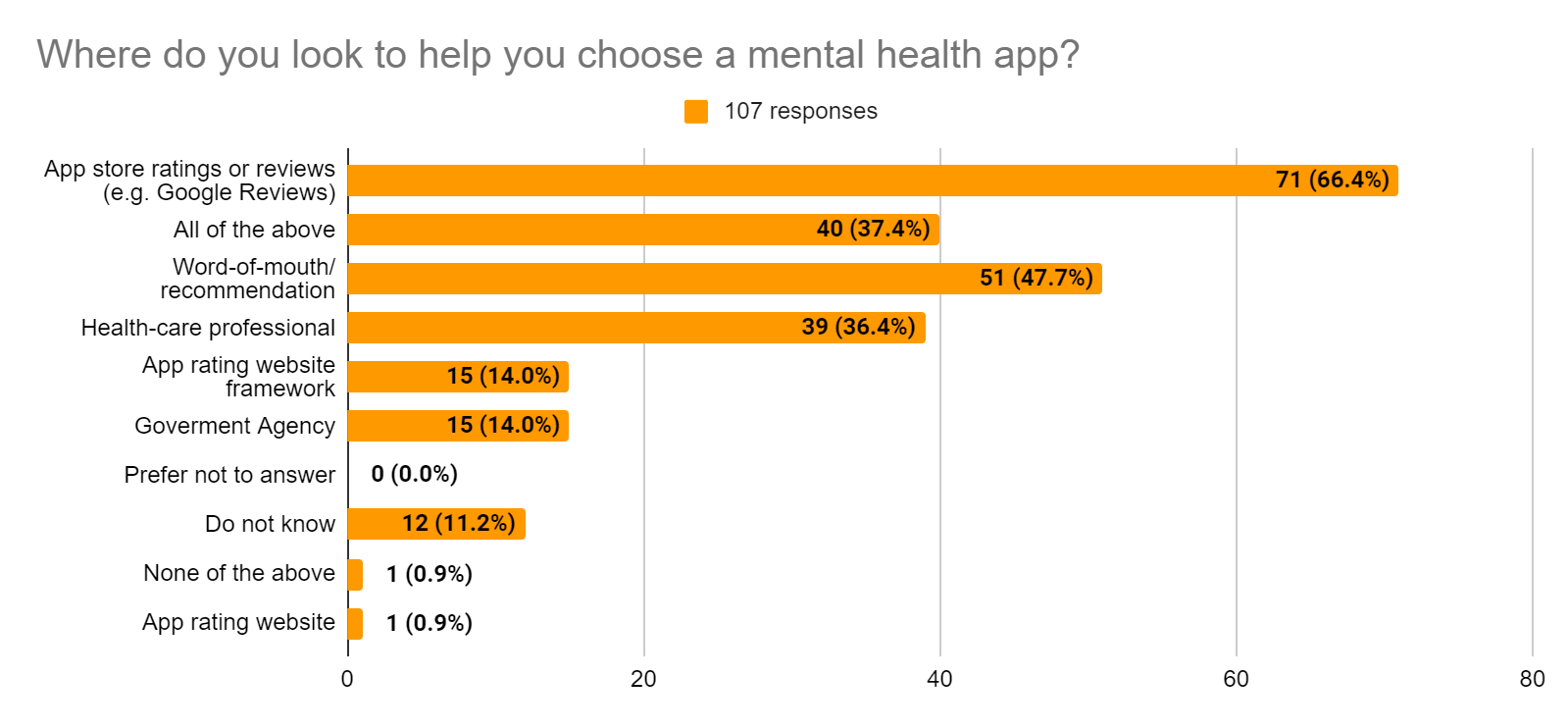
**

**
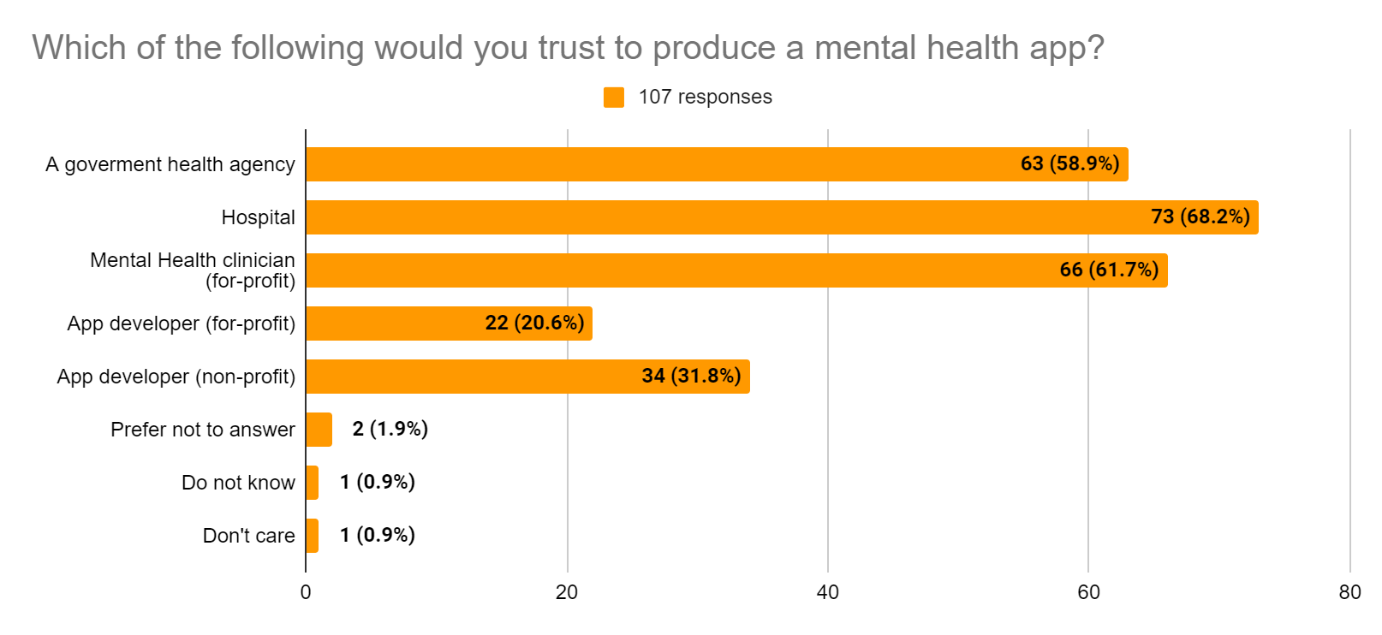
**

**
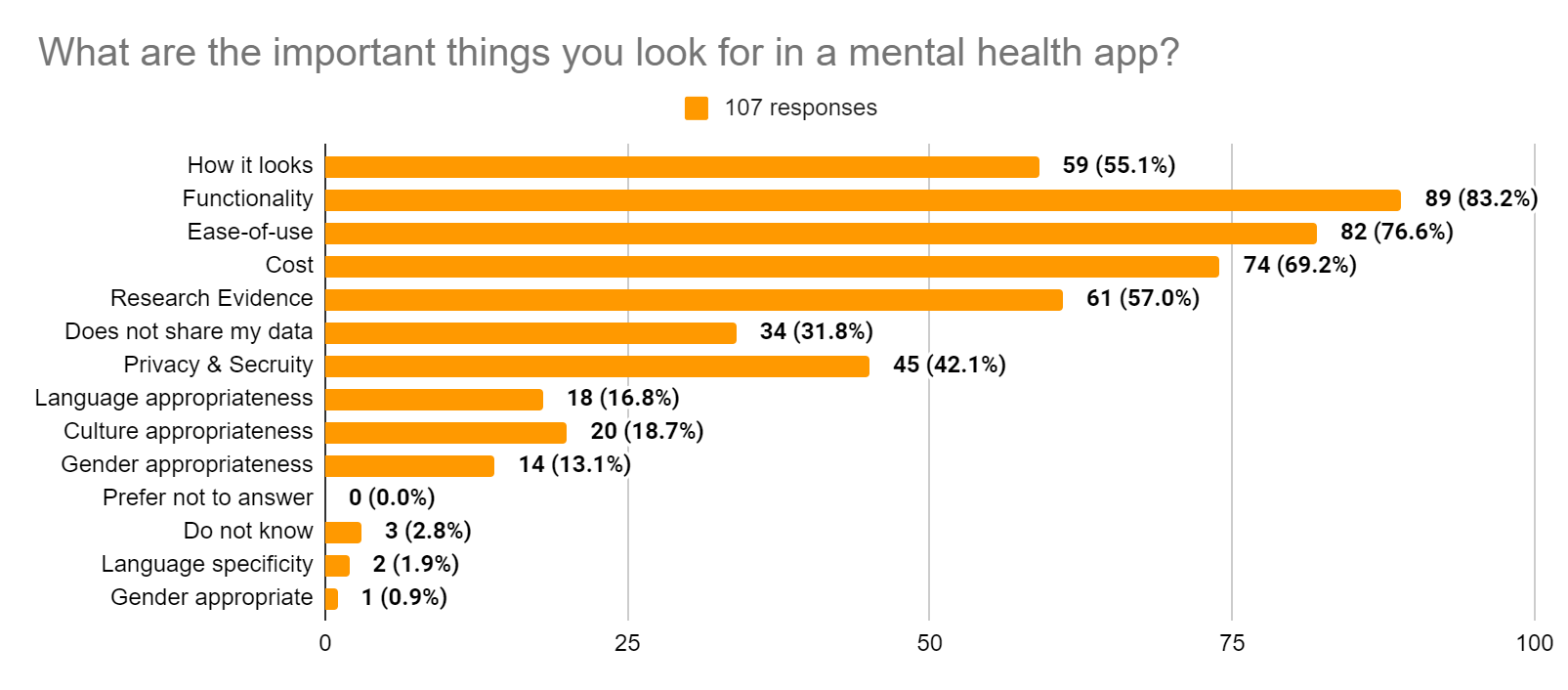
**

**
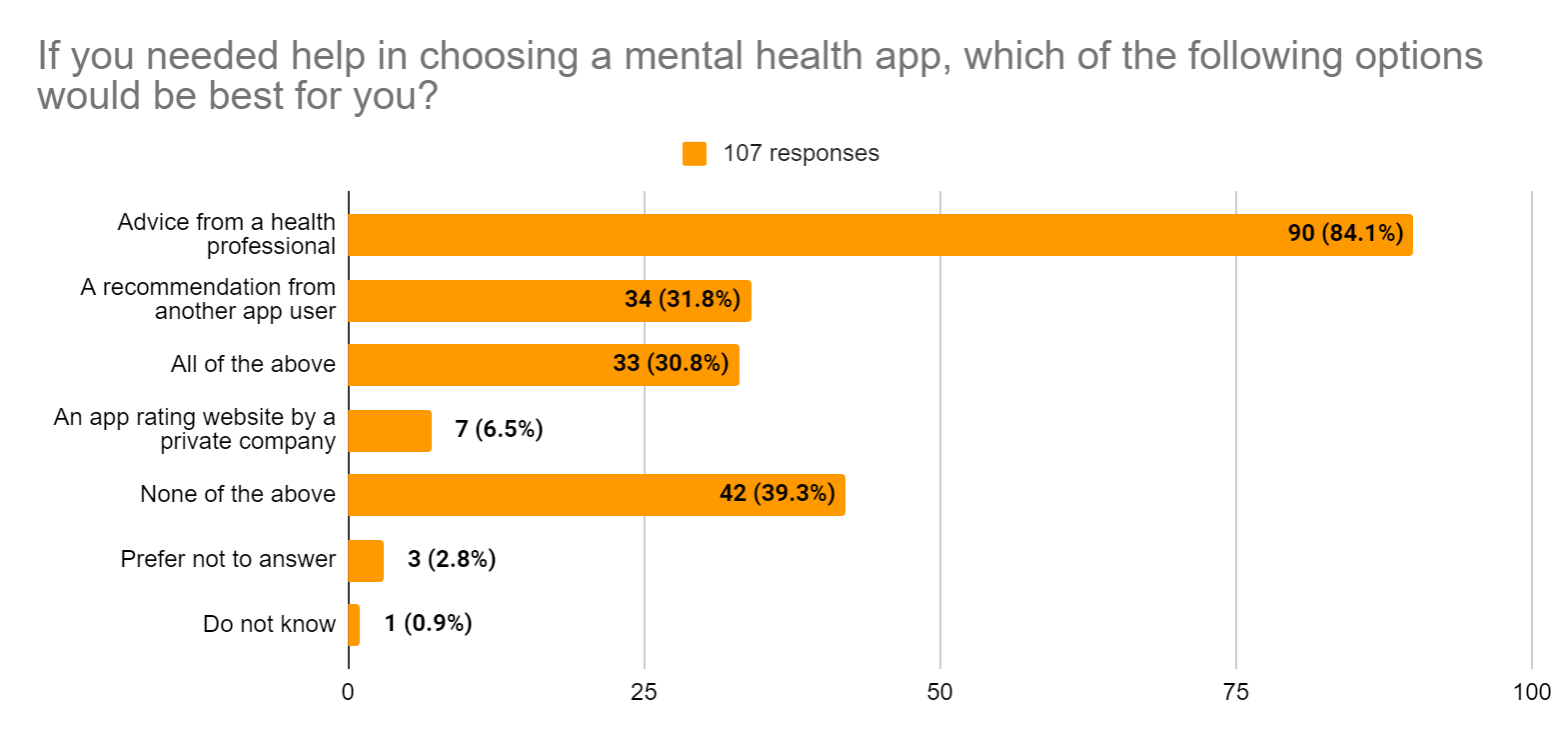
**

**
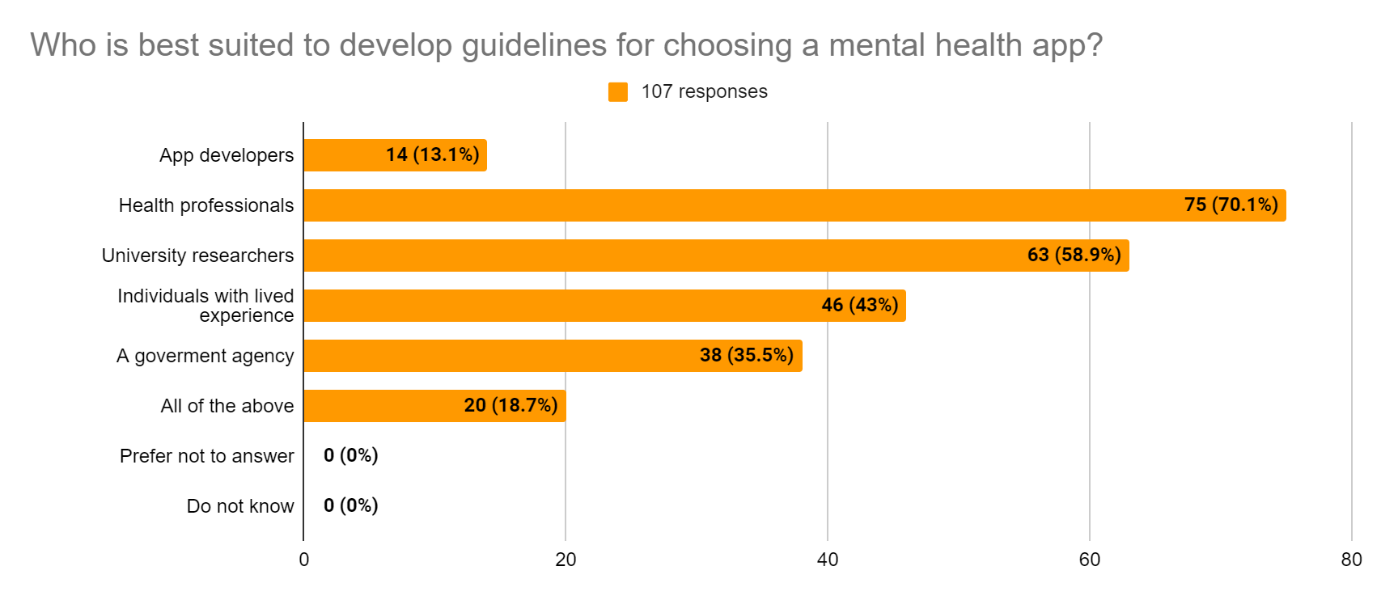
**
